# What is wellness? Investigating the importance of different domains of wellness among laypeople and experts: A survey study

**DOI:** 10.1177/14034948231217360

**Published:** 2024-01-12

**Authors:** Krista J. Kauppi, Eira T. Roos, Patrik T. Borg, Katarina S. Cantell, Paulus M. Torkki

**Affiliations:** 1 Department of Public Health, Faculty of Medicine, University of Helsinki, Finland; 2 Aava Medical Centre, Finland; 3 Aisti Health Ltd, Finland; 4 Adalyon Ltd, Finland

**Keywords:** Laypeople, wellness, well-being, model

## Abstract

**Aims:**

Lack of consensus on wellness has led to a vast number of different conceptualisations, which hinder international efforts to monitor individual-level wellness and social progress comparably. This study aimed to aid in the harmonisation of the concept by contributing to the scarce research on laypeople’s views on wellness. The study investigates whether the importance of different areas of wellness varies depending on age, gender, education or socio-economic position. Furthermore, considering that wellness models are often constructed by expert panels, this study aimed to shed light on how experts’ and laypeople’s views on wellness vary.

**Methods:**

Altogether, 1152 laypeople and 23 Finnish experts rated the importance of 61 systematic review-based wellness domains. Each domain received an ordinal number, which, together with the Mann–Whitney *U*-test or Kruskal–Wallis test, was used to examine the differences between the groups.

**Results:**

Thirteen wellness domains were found at the top of the lists, regardless of whether the results were analysed based on gender, age, education or socio-economic position. When looking at the priority order of different domains, we were able to identify several differences between the expert panel and laypeople.

**Conclusions:**

**To ensure the relevance of wellness models, it is vital to understand the areas that laypeople consider to be important for their comprehensive wellness. This study offers 13 domains that could be combined with an expert view on wellness and used as a starting point for creating a more comprehensive, inclusive and better-suited wellness instrument.**

## Introduction

The need to assess individuals’ wellness alongside GDP and other measures to examine social progress has been widely acknowledged. Increasing the level of wellness has been shown to have positive effects by increased productivity [1], reduced absenteeism and presenteeism [2], reduced hospital utilisation [3], lower health-care costs [3] and lower mortality [4]. Hence, there has been much critique regarding GDP as the sole measure of a nation’s prosperity [5]. This has led nations and organisations to establish programmes to incorporate aspects of wellness into their measurement instruments [6–8]. All of these measures aim to gain a more comprehensive view of how individuals are doing, but they tend to measure different areas and even use different terms for the same goals [6,7]. Consequently, although we aim to measure the same phenomena, our measures are not comparable. To compare how different political decisions and global and economic changes affect nations, it is vital to have a common understanding of what areas of wellness should be measured.

When identifying the areas of wellness to be measured, it is also important to take into consideration cultural and gender aspects [9]. However, while it has been common to examine how gender, age, education level and socio-economic status are associated with some specific areas of wellness, such as mental health, life satisfaction, overall health and life expectation [10–13], research into how these background variables affect the conceptualisation of wellness is lacking.

The absence of a consensus on the domains of wellness has led to a vast number of different conceptualisations developed using various methods but seldom taking into account the views of laypeople. A recent systematic review examined how different wellness models were created, and it noted that only one-third of the models were based on previous literature and often were developed by a group of scientists and later refined with population survey data [14]. Hence, laypeople are often not consulted about their views, but their data are used to develop the contents of measurement instruments through, for instance, factor analysis. This creates a risk of developing measurement instruments that might lack relevance to laypeople [15].

### Aims

This study investigated (a) what domains Finnish laypeople consider to be most important for comprehensive wellness; (b) how this varies depending on age, gender, education or socio-economic position; and (c) whether differences exist between Finnish experts and laypeople in the perceived importance of different domains of wellness. The study contributes to the scarce research around laypeople’s view on domains of wellness and offers health promotion researchers valuable information for development of measurement instruments that could be used to investigate the level of wellness of individuals and society.

## Methods

### Sample

The main group investigated in this study consisted of 1152 Finnish-speaking respondents from different parts of Finland (hereafter referred to as “laypeople”). As our focus was on the adult population, participants needed to be >18 years of age in order to answer the questionnaire. To investigate whether differences in opinion exist between experts and laypeople, another group was also included in the study. This group consisted of 23 experts who had been recruited for a separate Delphi study but who answered the same questionnaire in the first round of their process [16]. The demographics of both groups are presented in Table I. Data for the expert panel were collected in August 2021, and data for laypeople through an online questionnaire using a market research institute at the beginning of October 2021. The market research institute (Liidimedia Ltd) focuses on customer and market studies and used one of Finland’s largest consumer network platforms, where consumers receive a small reward for expressing their opinions.

**Table I.** Demographics of laypeople and the expert panel.

Laypeople		Expert panel	
Gender		Gender	
Male	445	Male	9
Female	700	Female	14
Other	6	Other	0
Do not want to say	1	Do not want to say	0
Area		Area	
Helsinki-Uusimaa	334	Helsinki-Uusimaa	17
Southern Finland	264	Southern Finland	2
Western Finland	268	Western Finland	2
Northern and Eastern Finland	286	Northe**r**n and Eastern Finland	2
Age (years)		Most describing background	
18–24	132	Personal trainer or physiotherapist	4
25–34	163	Work ability coach	4
35–44	175	Psychologist	4
45–54	205	Medical doctor	3
55–64	268	Nurse	4
>64	209	Human resources manager	4
Socio-economic background		Number of people with research background	5
Self-employed people	61	Professional experience from health care or wellness fields in years	21 (11)
Workers	515	Estimate of the familiarity of wellness field (average on scale 1–10)	9 (1)
Students	109		
Pensioners	306		
Unemployed	120		
Other	41		
Level of education			
Primary and lower secondary education (grades 1–9)	133		
Upper secondary education (high school or vocational education)	476		
Post-secondary non-higher vocational education	145		
Bachelor’s or equivalent	241		
Master’s or equivalent	144		
Doctoral or equivalent	13		

### Constructing the questionnaire

A systematic review was performed to gain a wide perspective of the different domains of wellness [14]. The review identified 44 different models and extracted 379 unique domains that clustered into 70 domain groups using thematic analysis. For this study, such general groups as ‘miscellaneous’ and ‘other physical’ were excluded, resulting in 61 domain groups. All participants were asked to evaluate the importance of these 61 literature-based wellness domains using a seven-point Likert scale, where 1=‘not important at all’, 4=‘neutral’ and 7=‘extremely important’). The questionnaire is presented in the Supplemental Material.

### Data analysis and synthesis

Data analysis was performed with the classification used in the questionnaire (e.g. age 18–24 years), with one exception in gender and education analyses. Participants who had stated their gender as ‘other’ or ‘does not want to say’ were excluded from these analyses due to the significantly small numbers of respondents (*n*=6 and *n*=1, respectively). Participants who had chosen a licentiate or PhD degree (*n*=13) were combined with the group that had achieved graduate degrees. To examine the differences among genders, age groups, education levels and socio-economic positions, mean and standard deviation were calculated using Microsoft Excel for all 61 domains. The results were filtered based on the mean from highest to lowest for each group, and an ordinal number was assigned to each domain. Furthermore, the Mann–Whitney *U*-test or Kruskal–Wallis test was performed using IBM SPSS Statistics for Window v28.0.0.0 (IBM Corp., Armonk, NY) to determine whether there were significant differences between the groups (*p*<0.05). Since the model created in the previous Delphi study [16] included 10 domains of wellness, in this review, we focused on the 10 first domains for each group. However, if the mean was the same or had only a difference of 0.01 to the next domain, the next domain was also included, resulting in some cases choosing 13 domains instead of 10.

## Results

Demographics of laypeople and the expert panel are presented in Table I. The sample of laypeople is geographically representative of Finland’s population but exhibits gender imbalance, with a greater proportion of women than men. Furthermore, relative to the Finnish population distribution, this sample is skewed towards younger age groups.

### Laypeople’s view on wellness

The most important domain was life satisfaction, with a mean value of 6.14, followed by sleep and recovery, with an average of 6.11. These were followed by mental health, safety, functioning, physical health and inner peace. Finns considered belief in a deity to be the least important domain of wellness, alongside spirituality. Furthermore, political environment, transcendence, genetics, cultural identity, gender identity or values and beliefs were at the bottom of the list. The average standard deviation (SD) was 1.3, and the SD increased towards the last domains, with one clear exception in the domain of anxiety and depression symptoms. The full results are shown in Table II.

**Table II.** Investigated wellness domains, their means, standard deviation and ordinal number based on the mean.

Domain	Average score	Difference to the next domain	Standard deviation	Ordinal number
Life satisfaction	6.14		1.1	1
Sleep and recovery	6.11	–0.03	1.1	2
Mental health	6.05	–0.06	1.2	3
Safety	6.03	–0.02	1.2	4
Functioning	6.02	–0.01	1.1	5
Physical health	5.90	–0.12	1.1	6
Inner peace	5.87	–0.03	1.2	7
Sense of worth	5.82	–0.05	1.1	8
Self-esteem	5.82	–0.01	1.1	9
Services and health care	5.81	0.00	1.2	10
Sense of humour	5.81	0.00	1.2	11
Self-responsibility	5.81	–0.01	1.1	12
Leisure	5.80	0.00	1.1	13
Nutrition	5.71	–0.09	1.2	14
Work–life balance	5.71	0.00	1.4	15
Lifestyle habits	5.70	–0.01	1.2	16
Stress management	5.70	0.00	1.1	17
Coping	5.70	0.00	1.2	18
Love	5.68	–0.01	1.4	19
Emotional management	5.67	–0.01	1.2	20
Energy	5.65	–0.02	1.2	21
Environment (nature and other)	5.63	–0.01	1.3	22
Emotional intelligence	5.58	–0.05	1.2	23
Cognitive health	5.56	–0.02	1.3	24
Meaningfulness	5.56	0.00	1.3	25
Self-awareness	5.56	–0.01	1.2	26
Sense of competence	5.54	–0.01	1.2	27
Health attitude	5.53	–0.01	1.2	28
Creativity and problem solving	5.51	–0.02	1.2	29
Exercise	5.49	–0.02	1.3	30
Emotional awareness	5.48	0.00	1.2	31
Identity	5.44	–0.04	1.2	32
Optimism	5.42	–0.02	1.3	33
Education and learning	5.41	–0.01	1.3	34
Financial and economic position	5.41	0.00	1.2	35
Social support	5.40	–0.01	1.3	36
Sense of control	5.39	–0.01	1.2	37
Social relationships	5.37	–0.02	1.3	38
Self-care and health behaviour	5.36	–0.01	1.2	39
Personal growth	5.32	–0.04	1.2	40
Social capabilities	5.29	–0.03	1.2	41
Positive and negative feelings	5.25	–0.03	1.2	42
Intellectual wellness	5.21	–0.04	1.2	43
Personality traits	5.13	–0.08	1.3	44
Community	5.11	–0.02	1.3	45
Autonomy	5.10	–0.02	1.4	46
Sex life	5.09	–0.01	1.4	47
Medical history	5.08	–0.01	1.4	48
Work	4.99	–0.10	1.5	49
Realistic beliefs	4.92	–0.07	1.3	50
Achieving in life	4.92	0.00	1.4	51
Anxiety and depression symptoms	4.92	0.00	1.7	52
Values and beliefs	4.86	–0.06	1.4	53
Body image	4.68	–0.18	1.5	54
Gender identity	4.65	–0.03	1.6	55
Cultural identity	4.64	–0.01	1.4	56
Genetics	4.59	–0.05	1.3	57
Transcendence	4.34	–0.25	1.3	58
Political environment	4.12	–0.22	1.5	59
Spirituality	3.72	–0.41	1.9	60
Belief in deity	2.83	–0.89	1.9	61

### Effect of background variables on laypeople’s views

**Figure 1.**
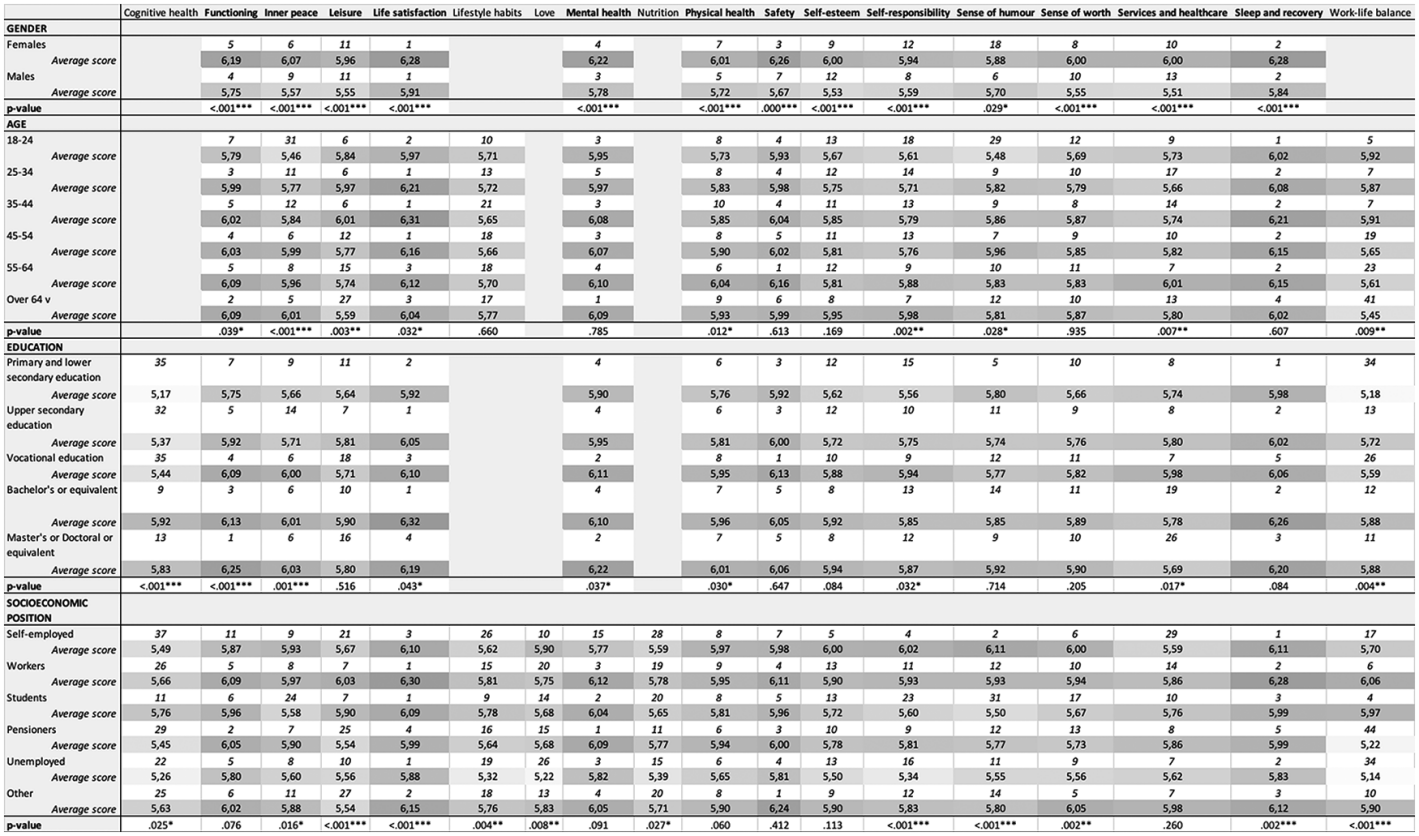
The most important wellness domains based on each background variable. We found 13 similar domains at the top of all lists. These domains are shown in bold. The first row indicates the ordinal number, and the second row shows the average score. *p < 0.05; **p < 0.01; *** *p* < 0.001.

Thirteen wellness domains were found at the top of lists, regardless of whether the results were analysed based on gender, age, education or socio-economic position (Figure 1). Arranged alphabetically, these were functioning, inner peace, leisure, life satisfaction, mental health, physical health, safety, self-esteem, self-responsibility, sense of humour, sense of worth, services and health care, and sleep and recovery.

Some significant differences (*p*<0.05) were found between genders (in 58/61 domains), age groups (29/61), education levels (38/61) and socio-economic positions (44/61). Women placed higher importance on safety, services and health care, and self-esteem, while men valued sense of humour and self-responsibility. The domains work–life balance and leisure were considered more important among respondents aged <45 years, with diminishing importance in higher age groups. The importance of the domains self-responsibility and inner peace was greater in higher age groups. The domains cognitive health, functioning and work–life balance received more emphasis from respondents with an undergraduate or graduate degree. The unemployed valued lifestyle habits, cognitive health, love, nutrition, work–life balance and self-responsibility less than the other groups.

### Differences between experts and laypeople

Both expert and laypeople panels valued sleep and recovery, functioning, life satisfaction, mental health, self-esteem and self-responsibility (Table III). Statistically significant differences were found in 16 (26%) domains. For instance, laypeople considered safety, inner peace, services and health care, sense of humour and leisure to be more important than experts did. Based on the ordinal number, experts in turn rated work–life balance, coping, meaningfulness, lifestyle habits, cognitive health and community more highly.

**Table III.** Full comparison of laypeople’s and experts’ views on different domains of wellness.

Domain	Average score: laypeople	Ordinal number: laypeople	Average score: expert	Ordinal number: experts	*p*
Life satisfaction	6.14	1	6.09	5	0.438
Sleep and recovery	6.11	2	6.52	1	0.116
Mental health	6.05	3	6.09	6	0.691
Safety	6.03	4	5.65	20	0.012
Functioning	6.02	5	6.09	4	0.808
Physical health	5.90	6	5.48	30	0.057
Inner peace	5.87	7	5.39	33	0.016
Sense of worth	5.82	8	5.52	28	0.209
Self-esteem	5.82	9	5.78	11	0.325
Services and health care	5.81	10	5.17	43	<0.001
Sense of humour	5.81	11	5.00	47	<0.001
Self-responsibility	5.81	12	5.70	16	0.457
Leisure	5.80	13	5.39	34	0.012
Nutrition	5.71	14	5.70	15	0.710
Work–life balance	5.71	15	6.13	3	0.179
Lifestyle habits	5.70	16	5.96	8	0.338
Stress management	5.70	17	5.57	26	0.279
Coping	5.70	18	6.13	2	0.110
Love	5.68	19	5.70	14	0.699
Emotional management	5.67	20	5.65	18	0.528
Energy	5.65	21	5.17	41	0.027
Environment (nature and other)	5.63	22	5.22	39	0.012
Emotional intelligence	5.58	23	5.17	40	0.025
Cognitive health	5.56	24	5.78	9	0.596
Meaningfulness	5.56	25	6.00	7	0.093
Self-awareness	5.56	26	5.26	37	0.082
Sense of competence	5.54	27	5.61	22	0.817
Health attitude	5.53	28	5.65	19	0.755
Creativity and problem solving	5.51	29	5.26	35	0.141
Exercise	5.49	30	5.70	13	0.602
Emotional awareness	5.48	31	5.61	21	0.817
Identity	5.44	32	5.57	25	0.952
Optimism	5.42	33	5.48	29	0.928
Education and learning	5.41	34	5.26	36	0.318
Financial and economic position	5.41	35	5.04	45	0.077
Social support	5.40	36	5.70	17	0.226
Sense of control	5.39	37	5.61	23	0.417
Social relationships	5.37	38	5.61	24	0.342
Self-care and health behaviour	5.36	39	5.52	27	0.696
Personal growth	5.32	40	5.17	42	0.310
Social capabilities	5.29	41	5.04	46	0.266
Positive and negative feelings	5.25	42	4.87	49	0.076
Intellectual wellness	5.21	43	4.26	55	<0.001
Personality traits	5.13	44	4.78	50	0.285
Community	5.11	45	5.78	10	0.013
Autonomy	5.10	46	5.70	12	0.049
Sex life	5.09	47	4.48	52	0.024
Medical history	5.08	48	4.43	53	0.023
Work	4.99	49	5.43	31	0.202
Realistic beliefs	4.92	50	4.43	54	0.055
Achieving in life	4.92	51	4.87	48	0.604
Anxiety and depression symptoms	4.92	52	5.39	32	0.237
Values and beliefs	4.86	53	5.09	44	0.599
Body image	4.68	54	5.22	38	0.111
Gender identity	4.65	55	4.65	51	0.855
Cultural identity	4.64	56	4.00	56	0.015
Genetics	4.59	57	4.00	57	0.007
Transcendence	4.34	58	3.70	60	0.021
Political environment	4.12	59	3.70	59	0.192
Spirituality	3.72	60	3.87	58	0.693
Belief in deity	2.83	61	3.26	61	0.173

## Discussion

As mainly experts’ views have been applied in wellness model development, we wanted to examine laypeople’s perspectives on the most important domains of wellness to be measured. Furthermore, in an attempt to take a step towards harmonising the concept of wellness, we also examined how this view varied between Finnish laypeople and a multidisciplinary expert panel. We found that despite background variables having numerous significant differences between genders, age groups, education levels and socio-economic status, we were able to identify 13 different domains of wellness that Finnish laypeople consider to be the most important ones. When these were compared with the experts’ opinions, some clear differences emerged, indicating that it is very important to also take laypeople’s opinions into account in model development.

Research on laypeople’s views regarding wellness has been scarce. While opinions of laypeople have been investigated in attempts to define the term ‘well-being’ [15,17], we could not find studies examining laypeople’s views on different prespecified areas of wellness or well-being. Our study disclosed that the most important wellness domains for Finnish laypeople were life satisfaction, sleep and recovery, mental health, safety, functioning, physical health, inner peace, sense of worth, self-esteem, services and health care, sense of humour, self-responsibility and leisure. This supports previous findings where laypeople raised life satisfaction, physical health, sense of humour, self-esteem and inner peace as factors relevant to well-being [15,17].

When comparing laypeople’s views with those of the expert panel, we could see numerous differences by looking at the ordinal number of different domains of wellness. Good examples are the domains of leisure, which was ranked highly by laypeople, and lifestyle habits, which was emphasised more by experts. Experts likely approach the rating of different domains from an evidence- or practice-based perspective, where education and years of experience affect their perception of wellness, while laypeople’s view is more affected by their cultural and social background and personal experiences. The perception of different areas of wellness is based on individual preferences and feelings instead of high-level scientific evidence. As what can be readily measured usually receives more attention, developing models solely based on expert opinion signifies that we risk measuring some areas of wellness that are irrelevant to laypeople. Previous literature has suggested multiple ways to involve laypeople in the decision-making process [18] and has shown positive effects when laypeople are included [19]. Considering that promoting laypeople to take part in policy decision making is one of the key action areas of the World Health Organization’s Member States through Rio Political Declaration on Social Determinants of Health, countries should carefully examine and measure whether they have mechanisms to support and engage society in policy development processes [20].

Whether considering the opinions of laypeople or experts, it is noteworthy that there are cultural and national differences in wellness, which highlights the importance of validating wellness measures in global settings. For instance, in our findings, Finns ranked belief in a deity and spirituality very low. This is aligned with the declining membership in churches in Finland [21], but the results would be very different had the survey been done in the Middle East or even the USA. Furthermore, Finns did not place much importance on the political environment. Considering that Finland is the most stable and has the best governance [22,23], Finns may take the political environment for granted, thus not perceiving its effect on wellness as high. However, it is noteworthy that the survey was conducted before the altercation in Ukraine. Otherwise, the results might be quite different.

Earlier research has found that age, gender and education affect our thinking and worldviews. Age has been shown to affect how we process information and the relative priority allocated to different goals in life [13], while gender affects behaviour and perception of wellness through, for instance, gender roles and societal expectations [24]. In Finland, this can still be seen as an occupational segregation, with, for example, women valuing safety more than men in this sample. The latter could be explained by women generally feeling lower levels of safety than men and, simultaneously, women engaging more in precautionary safety behaviours [25]. Furthermore, achieving a higher education has long-term effects on critical and conceptual thinking, attitudes and values, analytic skills, and the use of reason and evidence in addressing problems [26]. However, in our sample, we could only see slight differences in the responses between different groups. Hence, investigating whether these results are repeatable in a larger global sample is warranted.

What makes wellness research especially challenging is that the concept of wellness is continuously evolving. For instance, the domains highlighted by laypeople represent several topics associated with emotional, mental and social wellness more than the obvious physical aspects. These areas are gaining more value as we have entered an era where people struggle to find work–life balance in a changing world, where we have shifted from the Industrial Age into an era of automation and dramatic restructuring of professions. This finding thus seems logical, since the sample is mainly working-aged adults in Finland, where issues regarding wellness at work, work–life balance and mental health–related absences are at the forefront [27]. Furthermore, while both experts and laypeople rated the importance of sleep and recovery as high, this might not have been the case 15 years ago, as sleep research has since accelerated, and research results have become more visible to the general public [28].

Another difficult aspect of wellness is its dynamic nature, with different factors being interrelated and searching for a balance [9]. As the methodology of the paper focused on the ordinal view, we note that there are some limitations in terms of systems thinking. It may have been challenging for some respondents to place importance on different aspects of wellness and simultaneously consider their causal relationships. This can be seen in certain results. For instance, the category ‘social relationships’ was nowhere near the top of the lists, even though social connections have been shown to have a crucial effect on health and well-being [29].

When building wellness models, it is important to consider their dynamic, constantly evolving, subjective nature and attempt to understand the interrelations and the process of wellness. However, the only way forward is to start from somewhere, and an imperfect harmonised global measure of wellness is better than dozens of different incomparable ones. By combining both experts’ and laypeople’s views when developing complex measurement instruments, such as one for wellness, we would get closer to more inclusive, personalised, comprehensive and better-suited measures.

### Strengths and limitations

We identified some limitations that could have affected the review. This review focused solely on Finland. limiting the generalisability of the 13 discovered domains of wellness. However, we argue that an important aspect is including laypeople in the model development process, which can be considered generalisable. However, we highly recommend that fellow researchers conduct similar studies and preferably with a multinational focus to investigate the importance of different areas of wellness and how it varies depending on background variables. Furthermore, we did not collect information from the participants on whether they had a disability. As this can affect the perception of wellness, we recommend collecting this information in the future.

Furthermore, the basis of this study lies in a systematic review of the literature, which was strictly focused on wellness and well-being models that included at least physical, social and psychological domains. Thus, it is possible that the questionnaire did not include all possible domains of wellness. However, as the systematic review screened 3925 articles and used multiple databases and PRISMA guidelines, we consider the likelihood that some important domains would have been missed is low. Nonetheless, definitions of different domains would have been helpful. However, as there were no clear definitions for the domains, creating ones could have caused bias in the results. Considering the interesting finding of low priority of social connections, we recommend testing a different method to enable a more systems thinking approach.

Strengths of this study are that it focuses on a group that has seldom been investigated, includes 61 different wellness domains, also investigates the effect of background variables and compares the results with those of a multidisciplinary expert panel that used the same rating process of the same domains one month before, making the results very comparable.

### Implications for health promotion practice and research

It is vital to identify some universal domains of wellness that could be incorporated into global measurement instruments and to harmonise the measures to ensure that we measure the same thing and attain comparable results. There are already global instruments that provide global comparison statistics, such as the World Happiness Report, but we need to be able to agree on what should be measured for a comprehensive view of the wellness [30]. This study provides health promotion researchers with important insights into laypeople’s views on wellness and showcases why including laypeople in the model development process would be beneficial.

As this study focused solely on Finland, it is important to replicate it, preferably in a multinational setting, to determine how the importance of different domains of wellness, especially the top 13 domains, change. Combining experts’ and laypeople’s views would serve as a basis for a wellness framework that could be used to investigate how wellness data are linked to common burdens of individuals and societies such as burnout, generalised anxiety disorder and depression. Furthermore, the results of this study could provide a basis for forming and researching a dynamic systems model of wellness. We hope that our findings encourage health promotion researchers to include laypeople in the model development process and to create harmonised wellness measures for global use.

## Conclusions

This study aimed to harmonise the concept of wellness by investigating the areas of wellness that laypeople consider important and determining whether the ranking of these areas varies according to respondents’ age, gender, education level or socio-economic position. Furthermore, as wellness models are often developed by experts, we strove to raise discussion by comparing laypeople’s and experts’ evaluations of different areas of wellness. We were able to identify 13 wellness domains that could be combined with experts’ views and used as a basis for developing a wellness measurement instrument for further global validation. We encourage fellow researchers to continue the work towards harmonising the concept of wellness and attempting to build a more unified wellness instrument that could be used globally to reflect the level of wellness and social progress of different nations.

## Supplemental Material

sj-docx-1-sjp-10.1177_14034948231217360 – Supplemental material for What is wellness? Investigating the importance of different domains of wellness among laypeople and experts: A survey study Supplemental material, sj-docx-1-sjp-10.1177_14034948231217360 for What is wellness? Investigating the importance of different domains of wellness among laypeople and experts: A survey study by Krista J. Kauppi, Eira T. Roos, Patrik T. Borg, Katarina S. Cantell and Paulus M. Torkki in Scandinavian Journal of Public Health
