# Supplementary material for: What is wellness? Investigating the importance of different domains of wellness among laypeople and experts: A survey study
Source: Scand J Public Health. 2024 Jan 12;53(1):23–31. doi: 10.1177/14034948231217360 (PMC11742701; doi:10.1177/14034948231217360)
Supplement: sj-docx-1-sjp-10.1177_14034948231217360 – Supplemental material for What is wellness? Investigating the importance of different domains of wellness among laypeople and experts: A survey study [file sj-docx-1-sjp-10.1177_14034948231217360.docx]

**Supplement 1. Translated lay people questionnaire.**

Part 1: Background

1. Gender
   1. Female
   2. Male
   3. Other
   4. Do not want to say
2. Age
   1. 18-24
   2. 25-34
   3. 35-44
   4. 45-54
   5. 55-64
   6. Over 64
3. County
   1. Uusimaa
   2. Varsinais-Suomi
   3. Satakunta
   4. Kanta-Häme
   5. Pirkanmaa
   6. Päijät-Häme
   7. Kymenlaakso
   8. Etelä-Karjala
   9. Etelä-Savo
   10. Pohjois-Savo
   11. Pohjois-Karjala
   12. Keski-Suomi
   13. Etelä-Pohjanmaa
   14. Pohjanmaa
   15. Keski-Pohjanmaa
   16. Pohjois-Pohjanmaa
   17. Kainuu
   18. Lappi
   19. Ahvenanmaa
4. Education level
   1. Primary and lower secondary education (grades 1-9)
   2. Upper secondary education (high school or vocational education)
   3. Post-secondary non-higher vocational education
   4. Bachelor's or equivalent level
   5. Master's or equivalent level
   6. Doctoral or equivalent level
5. Socioeconomic position
   1. Self-employed persons
   2. Workers
   3. Students
   4. Pensioners
   5. Unemployed
   6. Other

Part 2: Estimating the importance of different areas of wellness

Please evaluate how important a role each of these domains plays in comprehensive wellness. *For instance: How important is the role of financial and economic position in comprehensive wellness or how important is the role of anxiety and depression symptoms in wellness? How much does community affect wellness?*

Rating will be done using a 7-point Likert scale as follows:

1 = Not important at all

2 = Not important

3 = Slightly not important

4 = Neutral

5 = Slightly important

6 = Important

7 = Extremely important

**Domains:**

1. Achieving in life
2. Autonomy
3. Anxiety and depression symptoms
4. Belief in deity
5. Body image
6. Cognitive health
7. Community
8. Coping
9. Creativity and problem solving
10. Cultural identity
11. Education and learning
12. Emotional awareness
13. Emotional intelligence
14. Emotional management
15. Energy
16. Environment, nature, and other
17. Exercise
18. Financial and economic position
19. Functioning
20. Gender identity
21. Genetics
22. Health attitude
23. Identity
24. Inner peace
25. Intellectual wellness
26. Leisure
27. Life satisfaction
28. Lifestyle habits
29. Love
30. Meaningfulness
31. Medical history
32. Mental health
33. Nutrition
34. Optimism
35. Personal growth
36. Personality traits
37. Physical health
38. Political environment
39. Positive and negative feelings
40. Realistic beliefs
41. Safety
42. Self-awareness
43. Self-care and health behaviour
44. Self-esteem
45. Self-responsibility
46. Sense of competence
47. Sense of control
48. Sense of humour
49. Sense of worth
50. Services and health care
51. Sex life
52. Sleep and recovery
53. Spirituality
54. Social capabilities
55. Social relationships
56. Social support
57. Stress management
58. Transcendence
59. Values and beliefs
60. Work
61. Work-life balance
